# Glottoplasty for vocal feminization in transgender women: position paper of the Brazilian Academy of Laryngology and Voice (ABLV)

**DOI:** 10.1016/j.bjorl.2026.101777

**Published:** 2026-03-09

**Authors:** Mateus Morais Aires, André Alencar Araripe Nunes, Érica Cristina Campos e Santos, Christiano de Giacomo Carneiro, Guilherme Simas do Amaral Catani, Adriana Hachiya, Erik Frota Haguette, Luciano Rodrigues Neves, Geraldo Druck Sant’ Anna, Marcos André de Sarvat

**Affiliations:** a Universidade de Pernambuco (UPE), Faculdade de Ciências Médicas, Recife, PE, Brazil; b Universidade Federal de Pernambuco (UFPE), Hospital das Clínicas, Recife, PE, Brazil; c Universidade Federal do Ceará (UFC), Fortaleza, CE, Brazil; d Universidade Federal da Bahia (UFBA), Hospital Universitário Prof. Edgard Santos, Salvador, BA, Brazil; e Universidade de São Paulo (USP) Bauru, Bauru, SP, Brazil; f Universidade Federal do Paraná (UFPR), Hospital das Clínicas, Curitiba, PR, Brazil; g Universidade de São Paulo (USP), Hospital das Clínicas, São Paulo, SP, Brazil; h Hospital Geral de Fortaleza (HGF), Fortaleza, CE, Brazil; i Universidade Federal de São Paulo (UNIFESP), Escola Paulista de Medicina, São Paulo, SP, Brazil; j Universidade Federal de Ciências da Saúde de Porto Alegre (UFCSPA), Porto Alegre, RS, Brazil; k Universidade Federal do Estado do Rio de Janeiro (UNIRIO), Rio de Janeiro, RJ, Brazil

**Keywords:** Voice disorders, Transgender persons, Consensus, Gender affirmation surgery

## Abstract

**Objective:**

To establish consensus-based recommendations for the indication, surgical technique, and perioperative care in glottoplasty for transgender women seeking vocal feminization.

**Methods:**

Ten otolaryngologists from distinct Brazilian academic institutions, all experienced in transgender voice surgery, contributed to this position paper. A modified Delphi method was applied, supported by a narrative literature review. Recommendations were classified using Oxford Centre for Evidence-Based Medicine levels.

**Results:**

Glottoplasty is indicated for transgender women experiencing vocal gender incongruence, regardless of their baseline fundamental frequency. Absolute and relative contraindications were defined, as well as recommended preoperative assessments such as videolaryngoscopy, acoustic voice analysis, and validated quality-of-life questionnaires. Perioperative management includes voice rest, individualized medication protocols, and post-operative voice therapy. Most recommendations are based on level IV and V evidence, reflecting the current paucity of prospective studies.

**Conclusion:**

This is the first Brazilian consensus on glottoplasty for transgender women, providing structured clinical guidance for indication, technical execution, and follow-up. Higher-level evidence is needed to strengthen future recommendations.

## Highlights

• Consensus-based recommendations using modified Delphi methodology.
• Clear surgical indications and contraindications were established.
• Comprehensive perioperative care recommendations are provided.
• Surgical indication prioritizes vocal gender incongruence, not acoustic measures.
• Most recommendations rely on low-grade evidence (case series and expert opinion).

## Introduction

Health care for transgender and gender-diverse individuals has intensified over the past decades across different medical specialties.^1^ This population comprises a significant number of individuals with particular needs due to stigmatization and discrimination.^2^ The exact prevalence of transgender identity remains unknown, with estimates of up to 5% of the global population.^3^ In Brazil, based on a representative sample of the adult population, transgender individuals represent 0.69% of the population, corresponding to an estimated 1,090,200 Brazilians identifying as transgender.^4^

The medical transition process for transgender patients is multidisciplinary, involving initial psychological evaluations, hormonal interventions, possible gender-affirming surgeries, and continuous mental health support.^1^ Voice plays a central role in gender perception. A voice incongruent with gender identity may limit full participation in social and professional activities, leading not only to difficulties in interpersonal relationships but also to triggering or exacerbating psychological disorders.^5^

The fundamental Frequency (F0) of vocal fold oscillation, perceived acoustically as pitch, is the main parameter used to identify voice gender. Generally, a voice is perceived as male when F0 is below 140 Hz and female when F0 is above 165 Hz. Other verbal and non-verbal behaviors – such as intonation, gesture, articulation, fluency, rhythm, and speech rate – also contribute to gender perception.^6^, ^7^, ^8^, ^9^

Transgender women who experience gender voice incongruence – that is, when they feel their voice does not reflect their identified gender – may seek vocal feminization strategies.^10^ Glottoplasty, described by Wendler in 1987 and popularized over the past decade, is currently the surgery of choice for vocal feminization. Its main goal is to raise vocal pitch by symmetrically shortening the vibrating portion of the vocal folds.^11^

This document aims to establish guidelines and recommendations, based on clinical expertise and literature review, for the management of transgender women seeking vocal feminization. Standardizing care, especially with emerging techniques, is essential to improving outcomes and safety.

## Methods

A panel composed of ten otolaryngologists, members of the Brazilian Academy of Laryngology and Voice (ABLV) and the Brazilian Association of Otolaryngology and Cervico-Facial Surgery (ABORL-CCF), affiliated with distinct tertiary university hospitals, contributed to the elaboration of this position paper. Eligibility criteria included active participation in surgical teams experienced in otolaryngological care for transgender patients, including glottoplasty.

Panelists completed an interactive form prepared by the coordinator of the document. Only the coordinator was unblinded to participants’ identities. Consensus statements were developed through a modified Delphi method, a systematic approach to achieve expert consensus in areas with scarce high-level evidence. Following collection and synthesis of responses, two virtual meetings were held for presentation of results, discussion, and consolidation of clinical consensus. In parallel, a narrative review of indexed publications in PubMed, LILACS, and Scopus databases was conducted. This study did not involve the collection, use, or analysis of individual patient data and was therefore exempt from submission to and approval by an Ethics Committee.

Levels of evidence and grades of recommendation were assigned to each statement, when applicable, according to the Oxford Centre for Evidence-Based Medicine classification^12^: Level I (evidence from randomized controlled trials), Level II (controlled studies without randomization), Level III (comparative, cohort, or case-control studies), Level IV (case series or descriptive studies), and Level V (expert opinion or case reports). Grades of recommendation were categorized as A (strong, high-level evidence), B (moderate), C (weak, limited evidence), and D (suggestion based on expert opinion or consensus).

## Results

### Patient selection and preoperative care

#### Surgical indication

- Transgender woman perceiving her voice as deep, inconsistent with the female gender, resulting in impaired quality of life.
- Transgender woman with an adapted voice who struggles to maintain a high-pitched voice in specific situations (particularly in emotional contexts and innate behaviors such as crying, laughing, or after coughing), reporting increased physical and cognitive effort.

The F0, assessed through acoustic voice analysis, should not be considered the sole criterion for surgical indication. The decision to undergo surgical voice modification must be regarded as an individual choice of each patient.

Level of evidence: III | Grade of recommendation: B

#### Preoperative requirements

- Minimum age of 18-years (according to Brazilian Federal Council of Medicine Resolutions nº 2.265/2019 and nº 2.427/2025).
- Medical diagnosis of gender incongruence.
- Recommended prior follow-up by a multiprofessional and interdisciplinary team, including psychiatrist and/or psychologist.
- Adequate expectations, with full understanding of the limitations of glottoplasty.
- Review and signature of the Informed Consent Form. The ABLV model form is suggested (Supplementary Appendix File 1).

Level of evidence: IV | Grade of recommendation: D

#### Absolute contraindications

- Severe psychotic disorders, severe personality disorders, intellectual disability, and severe global developmental disorders, as assessed by psychiatry.
- High surgical risk.

Level of evidence: IV | Grade of recommendation: D

#### Relative contraindications

- Singers and professional voice users. Although glottoplasty is effective in raising vocal pitch, it may negatively affect voice quality. Notably, reduction in vocal range (3.5 semitones or 86 Hz), maximum phonation time (0.7–1.1 seconds), and loudness (up to 50% of maximum intensity) may occur.^13^, ^14^, ^15^ These aspects must be thoroughly discussed preoperatively, requiring careful consideration for surgery in this subgroup.
- Pre-existing glottic lesions. Management should be individualized according to type and location, either before or concomitant with glottoplasty.^16^

Level of evidence: IV | Grade of recommendation: D

#### Preoperative multidisciplinary assessments

- Videolaryngoscopy with recording for medicolegal documentation and exclusion of contraindications. Stroboscopy is recommended for detailed glottic function analysis.^17^
- Acoustic voice recording to document objective vocal parameters preoperatively.^17^
- Speech therapy. Preoperative therapy is advisable, as it may facilitate verbal and non-verbal communication modifications and assist in the feminization process. Patients undergoing preoperative therapy tend to show better results due to greater control of vocal filters and improved adherence to postoperative rehabilitation. The number of sessions should be individualized.^13^^,^^18^, ^19^, ^20^, ^21^, ^22^, ^23^
- Endocrinological evaluation and hormone therapy. Endocrinological assessment and perioperative hormonal management are recommended. Specifically, testosterone suppression may influence vocal outcomes. According to the World Professional Association for Transgender Health (WPATH) guidelines, feminizing hormone therapy should combine estrogen with an antiandrogen.^1^

Level of evidence: V | Grade of recommendation: D

#### Voice-related quality of life questionnaires

- Trans Woman Voice Questionnaire (TWVQ). A validated self-perception instrument in Brazilian Portuguese.^24^, ^25^, ^26^, ^27^, ^28^

Level of evidence: III | Grade of recommendation: B

- Vocal Congruence Scale. A tool that assesses the degree of congruence between an individual’s voice and their gender identity, measuring vocal satisfaction. Cross-culturally adapted to Brazilian Portuguese.^29^^,^^30^

Level of evidence: V | Grade of recommendation: D

### Surgical technique

Glottoplasty is performed under general anesthesia with orotracheal intubation, using an endotracheal tube of appropriate caliber for laryngeal microsurgery. After placing the patient in the supine position and introducing the suspension laryngoscope, either the microscope or a rigid endoscope is used to visualize the surgical field. The procedure begins with de-epithelialization of the anterior portion of the free edge of the vocal folds, followed by suturing of this region with 2 or 3 stitches.^11^ In a variation of Wendler’s original technique, Kim proposed the Vocal Fold Shortening with Retrodisplacement of the Anterior Commissure (VFSRAC).^21^ In this technique, the thyroarytenoid muscle is sutured to the inner perichondrium of the subglottic portion of the thyroid cartilage, recreating the anterior commissure in a three-dimensional fashion. Variations of the surgical technique may be adopted according to surgeon preference.

Suture material, number of stitches, depth of the suture, craniocaudal extent of de-epithelialization, use of biological glue, and use of botulinum toxin do not appear to significantly affect vocal outcomes.^13^^,^^14^ Hospital discharge may occur on the same day or the following day, at the physician’s discretion. Importantly, glottoplasty requires good laryngeal exposure, with adequate visualization of the anterior commissure through the suspension laryngoscope. In cases where individual anatomic characteristics hinder sufficient exposure, the surgery may not be feasible, and alternative therapeutic options should be discussed.

#### Extent of the synechia

The intended synechia typically ranges from 30% to 60% of the length of the membranous vocal fold.^22^^,^^31^, ^32^, ^33^, ^34^ No direct correlation has been found between the extent of synechia and postoperative vocal outcomes.^35^ Ultimately, synechia formation depends on each patient’s individual healing process and adherence to postoperative care. No clinically significant airway restriction following glottoplasty has been reported; however, more extensive synechiae are theoretically associated with increased risk of dyspnea.

Level of evidence: IV | Grade of recommendation: C

#### Combination with other surgeries

There are no contraindications to performing glottoplasty in combination with other procedures. However, the decision should be individualized, considering the longer operative time and the potential for postoperative events related to the associated procedures – such as bleeding, pain, and coughing effort – that could compromise the glottoplasty outcome. The combination of glottoplasty with chondrolaryngoplasty (thyroid cartilage reduction) does not alter vocal outcomes.^16^^,^^36^

Level of evidence: IV | Grade of recommendation: C

#### Complications

Suture dehiscence occurs in 7%–14% of cases, being more frequent in smokers and in patients who do not strictly adhere to postoperative instructions.^13^^,^^14^ In these cases, clinical follow-up is recommended for approximately three months, the period necessary for the inflammatory process of the vocal folds to subside and functional results to be assessed. Surgical revision may be indicated when needed. In cases where only the most anterior suture dehisces, while the posterior portion remains intact, an anterior glottic gap may form. This is a rare complication with an undefined incidence, which, if symptomatic, may require reoperation for reconstruction of the synechia.^13^^,^^14^^,^^37^^,^^38^

Granuloma formation over the sutured area is estimated to occur in 5%–14% of cases.^37^^,^^39^, ^40^, ^41^, ^42^, ^43^ In persistent cases and according to clinical evaluation, management may include inhaled corticosteroids; if refractory and symptomatic, surgical excision might be necessary. Hypertrophic synechia, characterized by a scar longer or more fibrotic than usual, is a rare occurrence, with only five cases reported in the literature to date. This condition may result in severe dysphonia and dyspnea, sometimes requiring additional surgery.

Transient dyspnea due to glottic edema in the immediate postoperative period is a rare complication, with two cases reported.^44^ In most situations, it resolves with conservative measures, including inhaled and intravenous corticosteroids, adrenaline nebulization, and clinical monitoring. Finally, complications inherent to suspension laryngoscopy, such as dental trauma and soft tissue injuries, should also be considered.

Level of evidence: IV | Grade of recommendation: C

#### Vocal outcomes and phonatory repercussions

The final vocal result after glottoplasty cannot be predicted with accuracy, as outcomes vary widely, with F0 gains ranging from 3 to 112 Hz.^13^^,^^14^^,^^38^^,^^45^, ^46^, ^47^ Vocal pitch results from a complex interaction of physical and functional characteristics of the larynx, including control of respiratory support and subglottic pressure, modulation of tension and laryngeal elevation, and adjustment of the size and shape of resonance chambers. During the scarring process of the vocal folds, construction of a voice that represents each patient is gradually achieved with the aid of postoperative speech therapy, which facilitates vocal readaptation to the new glottic configuration.

Nonetheless, even when the synechia reaches the intended length, approximately 20% of patients fail to achieve a sufficiently high F0 for their voice to be perceived as female, due to anatomic-functional factors or, in some cases, a very low preoperative F0 ^13^^,^^14^^,^^38^^,^^45^, ^46^, ^47^ Persistent dysphonia, loss of vocal power, and muscle tension dysphonia may also occur, due to the creation of a fibrotic area with vibratory properties distinct from the physiologic vocal fold. These phonatory repercussions may be partially or completely managed with specialized speech therapy.

Level of evidence: III | Grade of recommendation: B

#### Vocal Fold Muscle Reduction (VFMR)

Also referred to as Laser Reduction Glottoplasty or Laser-Assisted Voice Adjustment (LAVA), this procedure consists of a laryngeal microsurgery aimed at reducing the mass of the vocal fold by vaporizing the thyroarytenoid muscle using laser or electrocautery.^32^^,^^48^, ^49^, ^50^ It may be indicated as a salvage procedure in patients who, after glottoplasty, present with an insufficiently high-pitched voice despite an adequately formed synechia. A waiting period of 4- to 6-months after glottoplasty is recommended before performing the VFMR, in order to complete vocal fold healing.

Level of evidence: IV | Grade of recommendation: C

### Postoperative care

#### Vocal rest

Absolute vocal rest for at least 10–15 days is required after surgery. Non-compliance with this recommendation is associated with an increased risk of suture dehiscence and the need for reoperation.^13^^,^^17^^,^^21^^,^^31^^,^^37^^,^^39^^,^^51^^,^^52^ In the following weeks, relative vocal rest and gentle vocal behavior are advised until initial synechia healing, typically around 30–40 days postoperatively.

Level of evidence: IV | Grade of recommendation: C

#### Medication

Medications such as antibiotics, analgesics, nonsteroidal anti-inflammatory drugs (NSAIDs), corticosteroids, proton pump inhibitors, and antitussives, among others, may be prescribed according to individualized medical evaluation and guidance.^13^^,^^38^^,^^45^, ^46^, ^47^

Level of evidence: IV | Grade of recommendation: C

#### Diet and physical activity

Dietary care should be individualized based on physician recommendations, with no need for strict dietary restrictions as routine. Physical activity should be avoided for two to four weeks, with adjustments according to each patient’s healing process.^13^^,^^38^^,^^47^

Level of evidence: V | Grade of recommendation: D

#### Videolaryngoscopy

Serial videolaryngoscopies should be performed to monitor healing until complete formation and stabilization of the synechia.^13^^,^^38^^,^^47^ Stroboscopy is recommended to evaluate vibratory patterns and detect glottic gaps.

Level of evidence: IV | Grade of recommendation: C

#### Vocal rehabilitation and postoperative speech therapy

Speech therapy should begin after completion of the absolute vocal rest period. On average, 12 sessions are recommended, with frequency and duration adjusted according to each patient’s progress and rehabilitation needs. Moderate to severe dysphonia is expected in the first weeks and months after the procedure. The final vocal outcome is usually reached around the sixth postoperative month.^23^^,^^36^^,^^42^^,^^53^, ^54^, ^55^, ^56^

Level of evidence: III | Grade of recommendation: B

#### Airway management and endotracheal intubation

A minimum interval of 3-months is recommended before undergoing subsequent elective surgery, in order to avoid rupture of the synechia during endotracheal intubation. Since the glottic area will be reduced, the use of smaller-diameter endotracheal tubes is advised whenever possible.^44^^,^^57^^,^^58^

In emergency intubation, ensuring adequate pulmonary ventilation is the priority. In such cases, a conventional adult-sized endotracheal tube may be necessary, even if it results in synechia rupture. If this occurs, surgical reintervention for reconstruction of the vocal synechia may be indicated after clinical stabilization.

Level of evidence: V | Grade of recommendation: D

## Discussion

This position paper by the Brazilian Academy of Laryngology and Voice (ABLV) proposes recommendations for glottoplasty as an intervention for vocal feminization in transgender women. Developed based on expert consensus and supported by a narrative literature review, it provides guidelines in a growing field. The use of a modified Delphi method ensures a structured and systematic approach in an area where high-level evidence remains scarce. The heterogeneity of participants, representing practices in distinct tertiary university hospitals across Brazil, offers a broad and representative national perspective.

Currently, the body of scientific evidence regarding glottoplasty remains limited, with predominance of case series and retrospective studies. Consequently, most recommendations are classified as Level IV or V evidence, with grades C or D. Only specific topics, such as surgical indication, use of validated quality-of-life questionnaires, vocal outcomes, and speech therapy, are supported by Level III evidence, allowing grade B recommendations. This scenario highlights the need for controlled clinical trials and prospective cohorts to improve recommendation quality.

## Conclusion

This document represents an essential first step toward standardizing glottoplasty practice in Brazil, providing clinical guidance based on the best available evidence and expert consensus. It underscores the need for higher-level scientific studies to strengthen future recommendations, identify relevant prognostic factors, and optimize surgical technique selection according to individual patient characteristics.

## ORCID ID

André Alencar Araripe Nunes: 0000-0001-5529-5369

Christiano de Giacomo Carneiro: 0009-0001-7473-2517

Adriana Hachiya: 0009-0003-2566-3660

Erik Frota Haguette: 0009-0002-7644-0021

Luciano Rodrigues Neves: 0000-0002-3088-3866

Marcos André de Sarvat: 0000-0002-1881-0437

## Funding

The authors have no funding or financial relationships to disclose.

## Data availability statement

The authors declare that all data are available in repository.

## Declaration of competing interest

The authors declare no conflicts of interest.
