# Supplementary material for: Glottoplasty for vocal feminization in transgender women: position paper of the Brazilian Academy of Laryngology and Voice (ABLV)
Source: Braz J Otorhinolaryngol. 2026 Mar 9;92(3):101777. doi: 10.1016/j.bjorl.2026.101777 (PMC12994021; doi:10.1016/j.bjorl.2026.101777)
Supplement: Supplementary file 1 [file mmc1.pdf]

TERMO DE CIÊNCIA E CONSENTIMENTO PARA GLOTOPLASTIA

Eu, ______________________________, RG _________ e CPF ____________, declaro que estou ciente de que a autorização para a realização de toda cirurgia pressupõe que eu esteja bem informada e esclarecida sobre as INDICAÇÕES e RISCOS do procedimento indicado, sendo que este visa contribuir para que se obtenha melhor HARMONIZAÇÃO VOCAL. Informo ter ciência e estou de acordo com o diagnóstico de TRANSGENERIDADE.

Fui satisfatoriamente informada de que a GLOTOPLASTIA representa uma proposta cirúrgica, definida caso a caso, para tornar a voz mais aguda, sendo realizada sob anestesia geral, através de acesso oral-faringo-laríngeo (interno), sem incisão cervical (corte no pescoço). Pode ocorrer inflamação local, hematoma ou infecção. Utilizarei a medicação que me será prescrita e seguirei os cuidados indicados. O grau de melhora da voz é imprevisível, ou seja, não pode ser precisado, e pode levar algumas semanas ou meses para se estabelecer. Minha voz não pode ser modulada ou acertada da forma exatamente como desejo, sei que não há como garantir que fique perfeitamente como gostaria. Entendi que a minha plena satisfação pode não ser atingida, e não há como reverter o procedimento para a laringe voltar a ser como agora (antes). O compromisso do médico consiste essencialmente em utilizar as técnicas usualmente recomendadas para tentar me oferecer uma voz mais aguda. A alta hospitalar pode acontecer no mesmo dia ou no dia seguinte ao procedimento. Poderá ocorrer algum incômodo na garganta ou no pescoço. Deverei manter repouso vocal e físico, seguir a dieta prescrita, não realizar esforços, não me expor a poeira, mofo, poluição, calor e frio. A liberação vai depender de minha evolução pós-operatória. Sei que febre, cansaço ou secreção podem representar complicações e deverão ser relatadas ao médico assistente. Eventual necessidade de reoperação pode ocorrer. Estou ciente de que a hormonioterapia pode ser fator favorável e que não a seguir pode prejudicar o resultado. Tenho ciência de que a área glótica será reduzida, podendo haver necessidade de posterior intubação com tubo de menor diâmetro.

Considero suficientes as informações e esclarecimentos prestados pelo médico otorrinolaringologista assistente, não me restando dúvidas ou questões, inclusive quanto a eventuais alternativas diagnósticas e terapêuticas, para minha tomada de decisão quanto a submeter-me à cirurgia ora proposta, e a todos os procedimentos que a incluem, inclusive anestesias ou outras condutas médicas que tal tratamento médico possa requerer, podendo o referido profissional valer-se do auxílio de outros profissionais da saúde. Estou ciente da necessidade de respeitar integralmente as instruções que me foram fornecidas pelo(a) médico(a), pois a sua não observância poderá acarretar riscos e efeitos colaterais. Declaro, igualmente, estar ciente de que o tratamento adotado não assegura a garantia de cura, e que a evolução pós-operatória e do tratamento podem obrigar o médico a modificar as condutas inicialmente propostas, sendo que, neste caso, fica o mesmo autorizado, desde já, a tomar providências necessárias para tentar a solução dos problemas surgidos, segundo seu julgamento, com o compromisso de me informar sobre tais modificações no primeiro momento possível.

Desta forma, levando em conta todas as informações prestadas, tendo as minhas dúvidas e questões devidamente esclarecidas, tomo a decisão de submeter-me ao procedimento ora proposto, agendado para o dia __ de _________ de 20__.

_______, __ de __________ de 20__.

....

Nome Médico(a)
